# ﻿Morphological and multi-gene phylogenetic analyses reveal *Nigrellomyces* gen. nov. and one new species in Pleurotheciaceae from China

**DOI:** 10.3897/mycokeys.122.164540

**Published:** 2025-09-18

**Authors:** Wang-ming Zhang, Xiao-yu Song, Wan-qing Xie, Xin-zhong Zhou, Juan Lu, Qin-ying Feng

**Affiliations:** 1 Beijing Jishuitan Hospital Guizhou Hospital, Guiyang 550014, China Beijing Jishuitan Hospital Guizhou Hospital Guiyang China

**Keywords:** Asexual morph, lignicolous freshwater fungi, phylogeny, Sordariomycetes, taxonomy

## Abstract

*Nigrellomyces* is introduced herein as a new genus to accommodate a novel asexual ascomycete, *Nigrellomyces aquaticus*, isolated from submerged decaying wood in freshwater habitats in Guizhou Province, China. Phylogenetic analyses based on a combined dataset of ITS, LSU, SSU, and *rpb*2 sequence data robustly place *Nigrellomyces* within the family Pleurotheciaceae (Pleurotheciales, Savoryellomycetidae), with strong statistical support. The genus is currently known only from its asexual morph, which is characterized by macronematous, mononematous, erect, unbranched, septate conidiophores; polyblastic, integrated, terminal or intercalary, sympodial proliferations; denticulate conidiogenous cells with swollen apices; and acrogenous, subglobose to globose, ovoid to obovoid, or ellipsoidal, aseptate, guttulate conidia. Morphological comparisons reveal that *Nigrellomyces* displays a cordana-like conidiogenesis that is distinct from other genera in Pleurotheciaceae, which typically exhibit acrodictys-, helicoön-, monodictys-, or dactylaria-like asexual morphs. This study provides a comprehensive morphological description, illustrations, and comparative interpretation of the new taxon. An updated phylogenetic backbone tree of Pleurotheciaceae is also presented, improving resolution of intergeneric relationships within the family. The discovery of *N. aquaticus* not only expands the taxonomic framework of Pleurotheciaceae but also contributes to a deeper understanding of the species richness, ecological roles, and biogeographic distribution of lignicolous freshwater fungi in Southwest China, particularly in the underexplored region of Guizhou Province.

## ﻿Introduction

Lignicolous freshwater fungi are a specialized group of fungi that colonize submerged or partially submerged woody substrates across a wide range of freshwater habitats, including streams, rivers, lakes, and reservoirs (Luo et al. 2004; Hyde et al. 2016; Calabon et al. 2022; Calabon et al. 2023). These fungi play an essential ecological role in the degradation of lignocellulosic materials, contributing significantly to the breakdown and recycling of organic matter. Through this decomposition process, they facilitate nutrient release and cycling, thus maintaining the ecological balance and supporting the biodiversity of freshwater ecosystems (Yuen et al. 1998; Bucher et al. 2004; Hyde et al. 2016; Calabon et al. 2023).

In recent decades, Asia has emerged as a global hotspot for lignicolous freshwater fungal research, with numerous studies focusing on the diversity and taxonomy of these fungi (Hyde et al. 2016; Su et al. 2016; Luo et al. 2019; Dong et al. 2020; Bao et al. 2021, 2023; Calabon et al. 2022, 2023; Shen et al. 2022b, 2024; Yang et al. 2023; Wang et al. 2024b; Xu et al. 2025b). In-depth investigations have been conducted, particularly in regions such as Guizhou, Hong Kong, Taiwan, and Yunnan in China, and Chiang Rai and Chiang Mai in Thailand. These regions have yielded a remarkable number of newly described taxa and have significantly expanded our understanding of the diversity and distribution of lignicolous freshwater fungi in tropical and subtropical habitats (Zhang et al. 2011; Jones et al. 2014; Hyde et al. 2016, 2021; Su et al. 2016; Bao et al. 2018, 2019, 2020, 2021, 2023; Luo et al. 2018b, 2019; Dong et al. 2020, 2021a, 2021b; Calabon et al. 2021, 2022, 2023; Shen et al. 2022a, 2022b, 2024; Xu et al. 2023, 2024b, 2025a, 2025b; Yang et al. 2023; Wang et al. 2024a, 2024b; Xiao et al. 2025). Among them, members of the Dothideomycetes and Sordariomycetes dominate lignicolous freshwater fungal communities, with ongoing research continuously revealing new species and higher-order lineages within these two classes (Su et al. 2016; Luo et al. 2019; Dong et al. 2020; Hyde et al. 2021; Calabon et al. 2022). Notably, the families Distoseptisporaceae, Fuscosporellaceae, Pleurotheciaceae, Savoryellaceae, Junewangiaceae, and Sporidesmiaceae of the Sordariomycetes constitute some of the most dominant and diverse groups of lignicolous freshwater fungi, encompassing many major taxa (Luo et al. 2019; Yang et al. 2023; Wang et al. 2024a, 2025; Xu et al. 2025b).

The family Pleurotheciaceae was established by Réblová et al. (2016), with *Pleurothecium* designated as the type genus. This family encompasses a diverse assemblage of fungi, including several species that inhabit submerged woody substrates in freshwater habitats (Réblová et al. 2016; Luo et al. 2018a; Dong et al. 2021b; Shi et al. 2021; Bao et al. 2022; Huang et al. 2022; Wang et al. 2024a; Xu et al. 2024a, 2025a). Both sexual and asexual morphs of Pleurotheciaceae have been reported, with the sexual morphs characterized by dark, papillate, perithecial, astromatic, immersed to superficial ascomata, unitunicate asci with a distinct non-amyloid apical annulus, and fusiform to ellipsoidal, septate, hyaline ascospores (Réblová et al. 2016; Luo et al. 2018a; Hyde et al. 2020). The asexual morphs of Pleurotheciaceae include species with diverse morphologies, comprising acrodictys-like (Yanna and Hyde 2002; Sadowski et al. 2012), helicoön-like (Dayarathne et al. 2019; Réblová et al. 2020), monodictys-like (Hyde et al. 2020), and dactylaria-like taxa (Réblová et al. 2016; Luo et al. 2018a).

In the latest update of “The 2024 Outline of Fungi and Fungus-like Taxa” compiled by Hyde et al. (2024), a total of 15 genera are accepted within the family Pleurotheciaceae, including *Adelosphaeria* Réblová, *Anapleurothecium* Hern.-Restr., R.F. Castañeda & Gené, *Coleodictyospora* Charles, *Dematipyriforma* L. Yan Sun, Hai Y. Li, Xiang Sun & L.D. Guo *Helicoascotaiwania* Dayar., Maharachch. & K.D. Hyde, *Melanotrigonum* Réblová, *Monotosporella* S. Hughes, *Neomonodictys* Y.Z. Lu, C.G. Lin & K.D. Hyde *Phaeoisaria* Höhn., *Phragmocephala* E.W. Mason & S. Hughes, *Pleurotheciella* Réblová, Seifert & J. Fourn., *Pleurothecium* Höhn., *Pseudosaprodesmium* X.G. Tian, K.D. Hyde & Tibpromma, *Saprodesmium* W. Dong & Doilom, and *Sterigmatobotrys* Oudem. The taxonomic placement of certain genera, particularly *Rhexoacrodictys*, has remained controversial. Initially, *Rhexoacrodictys* W.A. Baker & Morgan-Jones was placed in the order Savoryellales by Xia et al. (2017); subsequent molecular phylogenetic analyses by Luo et al. (2019) transferred *Rhexoacrodictys* to Pleurotheciales. Subsequent phylogenetic studies have confirmed the findings of Luo et al. (2019), providing robust support for the reassignment of *Rhexoacrodictys* (Dong et al. 2021b; Shi et al. 2021; Bao et al. 2022; Huang et al. 2022; Wang et al. 2024a, 2025).

Despite “The 2024 Outline of Fungi and Fungus-like Taxa” listing this genus under Savoryellaceae (Savoryellales), current phylogenetic evidence clearly indicates that *Rhexoacrodictys* is best placed in Pleurotheciaceae (Pleurotheciales), and its classification within this family is now widely accepted (Dong et al. 2021b; Shi et al. 2021; Bao et al. 2022; Huang et al. 2022; Wang et al. 2024a, 2025). Furthermore, *Obliquifusoideum* W. Dong & Doilom was recently included in Pleurotheciaceae by Wang et al. (2024a), based on both morphological characteristics and phylogenetic evidence. In the other study, based on morphological characteristics and DNA sequence data, *Saprodesmium* was treated as a synonym of *Rhexoacrodictys* (Wang et al. 2025). To date, the family Pleurotheciaceae comprises sixteen genera and more than 100 species, which are primarily saprobic on a variety of decaying plant substrates in both freshwater and terrestrial habitats (Réblová et al. 2016; Luo et al. 2018a; Dong et al. 2021b; Shi et al. 2021; Bao et al. 2022; Huang et al. 2022; Wang et al. 2024a; Xu et al. 2024a, 2025a).

During a recent investigation of lignicolous freshwater fungi in Guizhou Province, China, a previously undescribed cordana-like species was isolated. Phylogenetic analyses based on a combined dataset of ITS, LSU, SSU, and *rpb*2 gene regions revealed that the new collection formed a well-supported and distinct clade within the family Pleurotheciaceae. Based on a comprehensive assessment integrating detailed morphological observations and multi-locus phylogenetic evidence, we introduce a new genus to accommodate this unique taxon. This study provides a thorough taxonomic treatment of the new taxa, including detailed morphological descriptions, high-quality illustrations, and comparative analyses with closely related taxa. In addition, an updated phylogenetic backbone tree of Pleurotheciaceae is presented, offering insights into the relationships among genera within the family. Our findings contribute not only to the taxonomic richness of lignicolous freshwater fungi in Guizhou Province but also enhance our broader understanding of fungal diversity associated with freshwater habitats in southwest China.

## ﻿Materials and methods

### ﻿Sample collection and specimen examination

Lignicolous substrates (decaying wood) were collected from a stream in Baiyun District, Guiyang City, Guizhou Province, China. Samples were taken to the laboratory in plastic bags and labeled with collection details, including locality, habitat, and date (Rathnayaka et al. 2024). Samples were cultured in plastic boxes lined with moistened tissue at room temperature for 1–2 weeks (Yang et al. 2023). The samples were examined using a stereomicroscope (SMZ 745, Nikon, Japan). Micromorphological characters were captured using a Nikon EOS 90D digital camera attached to an ECLIPSE Ni compound microscope (Nikon, Japan). Measurements of conidiophores, conidiogenous cells, and conidia were carried out using the Tarosoft (R) Image Frame Work program.

### ﻿Isolation and material deposition

Single-spore isolation was performed following the method described by Senanayake et al. (2020). The germinated conidia were aseptically transferred to fresh potato dextrose agar (PDA; Oxoid, CM0139) and incubated at room temperature for 41 days. Morphological characteristics of the fungal mycelium on PDA, including color, shape, size, margin, and elevation, were documented. Dried fungal specimens were deposited in the Herbarium of Guizhou Academy of Agricultural Sciences (Herb. GZAAS), Guiyang, China. Pure cultures were deposited at the Guizhou Culture Collection (GZCC), Guiyang, China. Descriptions of the new taxa were uploaded to the Faces of Fungi webpage following the guidelines of Jayasiri et al. (2015). The new species were registered in the MycoBank database (https://www.mycobank.org/), and MycoBank numbers were obtained.

### ﻿DNA extraction, PCR amplification, and sequencing

Fresh fungal mycelia grown on PDA were scraped using sterilized scalpels. Genomic DNA was extracted using the Biospin Fungus Genomic DNA Extraction Kit (BioFlux, China), following the manufacturer’s protocol. The primer pairs ITS5/ITS4 (White et al. 1990), LR0R/LR5 (Vilgalys and Hester 1990), NS1/NS4 (White et al. 1990), and fRPB2-5F/fRPB2-7cR (Liu et al. 1999) were used to amplify the ITS, LSU, SSU, and *rpb*2 regions, respectively. PCR amplification was performed in a 25 μL reaction volume, consisting of 13.5 μL of 2× Taq PCR Master Mix (China; containing Taq DNA polymerase, dNTPs, MgCl_2_, and reaction buffer), 1 μL of each primer, 1 μL of template DNA, and 8.5 μL of ddH_2_O. The polymerase chain reaction (PCR) conditions employed were in accordance with the reaction conditions outlined by Wang et al. (2024a). The PCR products were purified and sequenced by Sangon Biotech (Shanghai, China) Co., Ltd.

### ﻿Phylogenetic analyses

BioEdit v. 7.0.5.3 (Hall 1999) and SeqMan v. 7.0.0 (Swindell and Plasterer 1997) were used to check and assemble the newly generated sequences. Sequences obtained in this study (Table 1) were deposited in the NCBI GenBank database (https://blast.ncbi.nlm.nih.gov/Blast.cgi). Multiple sequence alignments for each locus dataset were performed using MAFFT v.7.473 (https://mafft.cbrc.jp/alignment/server/, Katoh et al. 2019) and visually inspected in AliView (Larsson 2014). The LSU and ITS alignments were trimmed using trimAl v1.2rev59 (Capella-Gutiérrez et al. 2009) and subsequently merged using SequenceMatrix v1.7.8 (Vaidya et al. 2011).

**Table 1.** Taxa used in this study, along with their corresponding GenBank accession numbers.

Taxon	Strain/Specimens	GenBank accession numbers			
		ITS	LSU	SSU	*rpb*2
*Adelosphaeria catenata*	CBS 138679^T^	KT278721	KT278707	KT278692	KT278743
*Anapleurothecium botulisporum*	FMR 11490^T^	KY853423	KY853483	–	–
*Canalisporium exiguum*	SS 00809	GQ390296	GQ390281	GQ390266	HQ446436
*Canalisporium grenadoideum*	SS 03615	–	GQ390267	GQ390252	HQ446420
*Canalisporium pulchrum*	SS 03982	GQ390292	GQ390277	GQ390262	HQ446432
*Coleodictyospora muriformis*	MFLUCC 18-1243^T^	MW981642	MW981648	MW981704	–
*Coleodictyospora muriformis*	MFLUCC 18-1279	MW981643	MW981649	MW981705	–
*Conioscypha lignicola*	CBS 335.93	–	AY484513	JQ437439	JQ429260
*Conioscypha minutispora*	CBS 137253	–	MH878131	–	–
*Dematipyriforma aquilaria*	CGMCC 3.17268^T^	KJ138621	KJ138623	KJ138622	–
*Dematipyriforma muriformis*	MFLU 21-0146^T^	OM654773	OM654770	–	–
*Dematipyriforma nigrospora*	MFLUCC 21-0096^T^	MZ538524	MZ538558	–	–
*Dematipyriforma nigrospora*	MFLUCC 21-0097	MZ538525	MZ538559	MZ538574	MZ567113
*Helicoascotaiwania farinosa*	DAOM 241947	JQ429145	JQ429230	–	–
*Helicoascotaiwania lacustris*	CBS 145963^T^	MN699399	MN699430	MN699382	MN704304
*Helicoascotaiwania lacustris*	CBS 146144	MN699401	MN699432	MN699384	MN704306
*Melanotrigonum ovale*	CBS 138743^T^	KT278724	KT278709	KT278696	KT278745
*Melanotrigonum ovale*	CBS 138742	KT278723	KT278708	KT278695	KT278744
*Monotosporella setosa*	HKUCC 3713	–	AF132334	–	–
*Neoascotaiwania fusiformis*	MFLU 15-1156^T^	MG388215	NG_057114	–	–
*Neoascotaiwania fusiformis*	MFLUCC 15-0625	–	KX550894	KX550898	–
*Neomonodictys aquatica*	KUNCC 21-10708^T^	MZ686200	OK245417	–	–
*Neomonodictys muriformis*	MFLUCC 16-1136^T^	MN644509	MN644485	–	–
*Nigrellomyces aquaticus*	GZCC 25-0630^T^	PV871229	PV871235	–	PV872880
*Nigrellomyces aquaticus*	GZCC 25-0631	PV871230	PV871236	–	PV872881
*Obliquifusoideum guttulatum*	MFLUCC 18-1233^T^	MW981645	MW981650	MW981706	–
*Obliquifusoideum triseptatum*	CGMCC 3.27014^T^	PP445243	PP049503	PP049521	PP068779
*Phaeoisaria motuoensis*	KUNCC 10410T	OP626333	OQ947034	OQ947036	–
*Phaeoisaria motuoensis*	KUNCC 10450	OQ947032	OQ947035	OQ947037	–
*Phaeoisaria obovata*	CGMCC 3.27015^T^	PP049488	PP049504	PP049522	PP068788
*Phaeoisaria obovata*	KUNCC 23-15598	PP049489	PP049505	PP049523	PP068784
*Phaeoisaria sedimenticola*	CGMCC3.14949^T^	JQ074237	JQ031561	–	–
*Phaeoisaria sedimenticola*	KUNCC 23-14648	PP049490	PP049506	PP049524	PP068783
*Phaeoisaria synnematica*	NFCCI 4479^T^	MK391494	MK391492	–	–
*Phaeoisaria synnematica*	KUNCC 23-16619	PP049493	PP049509	PP049527	PP068787
*Phragmocephala stemphylioides*	KAS 4277	KT278730	KT278717	–	–
*Pleurotheciella aquatica*	MFLUCC 17-0464^T^	MF399236	MF399253	MF399220	MF401405
*Pleurotheciella brachyspora*	CGMCC 3.25435^T^	OR589321	OR600969	PP049532	PP068773
*Pleurotheciella fusiformis*	MFLUCC 17-0113^T^	MF399233	MF399250	MF399218	MF401403
*Pleurotheciella fusiformis*	MFLUCC 17-0115	MF399232	MF399249	MF399217	MF401402
*Pleurotheciella guttulata*	KUMCC 15-0442	MF399239	MF399256	MF399222	MF401408
*Pleurotheciella guttulata*	KUMCC 15-0296^T^	MF399240	MF399257	MF399223	MF401409
*Pleurotheciella longidenticulata*	CGMCC 3.27018^T^	PP049496	PP049513	PP049531	PP068776
*Pleurothecium aquaticum*	MFLUCC 17-1331^T^	MF399245	MF399263	–	–
*Pleurothecium aquaticum*	MFLUCC 21-0148	OM654775	OM654772	OM654807	–
*Pleurothecium aquisubtropicum*	GZCC 21-0670^T^	OM339436	OM339433	–	–
*Pleurothecium pisiformis*	KUNCC 24-19085^T^	PV264837	PV264846	PV335238	–
*Pleurothecium pulneyense*	MFLUCC 16-1293	–	MF399262	MF399228	MF401414
*Pleurothecium semifecundum*	CBS 131482	JQ429158	JQ429239	JQ429253	–
*Pleurothecium semifecundum*	CBS 131271^T^	JQ429159	JQ429240	JQ429254	JQ429270
*Pseudosaprodesmium cocois*	MFLU 23-0225^T^	OR438401	OR438864	OR458363	–
*Pseudosaprodesmium cocois*	GZAAS 23-0588	OR438402	OR438865	OR458364	–
*Rhexoacrodictys fimicola*	HMAS 43690	KU999957	KX033550	KX033519	–
*Rhexoacrodictys fimicola*	HMAS 47737	KU999960	KX033553	KX033522	–
*Rhexoacrodictys fimicola*	MFLUCC 18-0340	OM654774	OM654771	OM654806	–
*Rhexoacrodictys melanospora*	KUNCC 22-12406^T^	OP168085	OP168087	OP168088	OP208807
*Rhexoacrodictys melanospora*	KUNCC 22-12411	OP168093	OP168101	OP168099	OP208808
*Saprodesmium dematiosporium*	KUMCC 18-0059^T^	MW981646	MW981647	MW981707	–
*Sterigmatobotrys macrocarpa*	PRM 915682	JQ429153	GU017317	JQ429255	–
*Sterigmatobotrys rudis*	DAOM 229838	JQ429152	JQ429241	JQ429256	JQ429272

Note: “^T^” indicates ex-type strains. Newly generated sequences are in bold black. “-” indicates the unavailable data in GenBank.

Maximum likelihood (ML) analysis was conducted using the IQ-TREE web server (http://iqtree.cibiv.univie.ac.at/) based on Bayesian Information Criteria (BIC) (Nguyen et al. 2015). The substitution model was automatically selected by the server. Bayesian inference (BI) analysis was performed using MrBayes on XSEDE (3.2.7a) via CIPRES Science Gateway (Stamatakis 2014). The aligned FASTA file was converted to NEXUS format using AliView (Larsson 2014). The best-fit evolutionary model for each dataset was determined using MrModeltest v. 2.3.10 (Nylander 2004). The GTR+G+I substitution model was selected for LSU, ITS, and *rpb*2, whereas the K80+I+G model was chosen for SSU. Posterior probabilities (BYPP) were determined based on Bayesian Markov chain Monte Carlo (BMCMC) sampling (Huelsenbeck and Ronquist 2001). Four simultaneous Markov chains were run for 10,000,000 generations, and trees were sampled every 1,000 generations. The burn-in phase was set at 25%, and the remaining trees were used to calculate posterior probabilities.

Phylogenetic trees were visualized using FigTree v. 1.4.4 and further edited in PowerPoint. The photo plate was prepared using Adobe Photoshop CS6 software (Adobe Systems, USA).

### ﻿Phylogenetic analysis results

**Figure 1.**
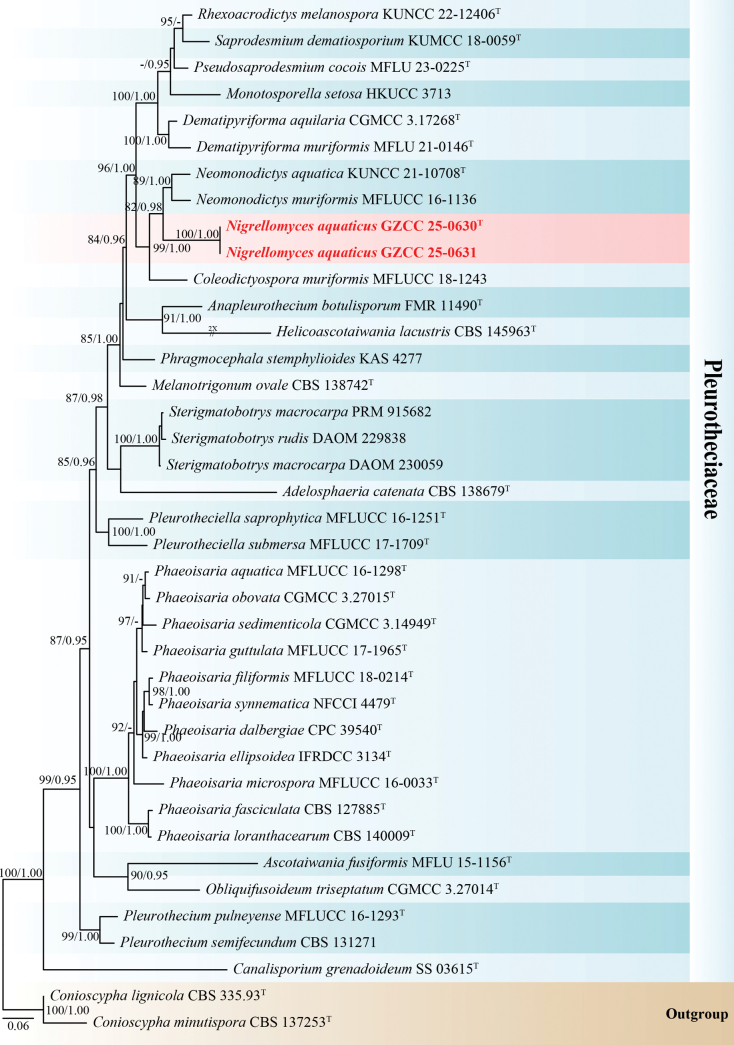
Phylogenetic tree generated from ML analysis based on the combined LSU, ITS, SSU, and *rpb*2 sequence data. Bootstrap support values for maximum likelihood greater than 75% (ML left) and Bayesian posterior probabilities ≥ 0.95 (BYPP right) are shown at the nodes. The tree was rooted with *Conioscypha lignicola* (CBS 335.93) and *C. minutispora* (CBS 137253). Ex-type strains are denoted with “^T,^” and newly isolated strains are in bold red fonts.

The single-locus and multi-locus phylogenetic analyses (LSU, ITS, SSU, and *rpb*2) were implemented to elucidate the phylogenetic position of the two new strains. The concatenated sequence matrix comprised 3,102 characters (LSU: 1–826, ITS: 827–1,371, SSU: 1,372–2,143, and *rpb*2: 2,144–3,102) across 60 taxa. Maximum likelihood (ML) and Bayesian inference (BI) analyses were conducted on the concatenated datasets of LSU, ITS, SSU, and *rpb*2, both yielding similar tree topologies. Fig. 1 presents the best-scoring ML tree, which had a final log-likelihood value of –25777.951173.

Based on the multi-gene phylogenetic tree (Fig. 1), our collections represent a new genus and a novel species within the family Pleurotheciaceae (Pleurotheciales, Sordariomycetes). Our isolates (GZCC 25-0630 and GZCC 25-0631) formed a sister clade to the clade comprising *Neomonodictys aquatica* (KUNCC 21-10708) and *N. muriformis* (MFLUCC 16-1136) with weak support.

## ﻿Taxonomy

### ﻿ Nigrellomyces

Taxon classificationFungiPleurothecialesPleurotheciaceae

W.M. Zhang & Q.Y. Feng gen. nov.

7F7424D8-99EB-5146-AFCA-7FC21943D0B4

904235

#### Etymology.

“*Nigrellomyces*” refers to the small, dark-colored conidia characteristic of this genus.

#### Description.

***Saprobic*** on decaying wood submerged in a freshwater stream. **Asexual morph: *Colonies*** superficial, effuse, hairy, brown to black, scattered, with glistening conidial masses at apex. ***Mycelium*** partly superficial, partly immersed, consisting of branched, septate, smooth, smooth-walled, hyaline to pale brown hyphae. ***Conidiophores*** macronematous, mononematous, solitary, erect, unbranched, septate, straight or flexuous, cylindrical, slightly constricted at septa, dark brown, becoming pale brown to brown towards the apex. ***Conidiogenous cells*** polyblastic, integrated, terminal or intercalary sympodial proliferations, sometimes denticles, cylindrical, reniform, swollen at the top, pale brown to brown. ***Conidia*** acrogenous, subglobose to globose, ovoid to obovoid, or ellipsoidal, aseptate, occasionally forming chains in water, guttulate, subhyaline to brown or black, smooth-walled. **Sexual morph**: Undetermined.

#### Type species.

*Nigrellomyces aquaticus* W.M. Zhang & Q.Y. Feng

#### Notes.

Morphologically, *Nigrellomyces* can be readily distinguished from other genera in Pleurotheciaceae by its polyblastic, reniform conidiogenous cells with curved apices and subglobose to globose, ovoid to obovoid, or ellipsoidal, aseptate conidia that occasionally form chains in water. Phylogenetically, *Nigrellomyces* forms a distinct clade within Pleurotheciaceae, supporting its recognition as a new genus. Herein, we establish the genus *Nigrellomyces* to accommodate a new species, *N. aquaticus*, which is designated as the type species based on both molecular evidence and its distinctive conidial morphology.

### ﻿ Nigrellomyces aquaticus

Taxon classificationFungiPleurothecialesPleurotheciaceae

W.M. Zhang & Q.Y. Feng sp. nov.

7F555DA3-023B-5642-BD3C-D56CA69F9FEB

904236

Fig. 2

#### Etymology.

“*aquaticus*’’ refers to the aquatic habitat of this fungus.

**Figure 2.**
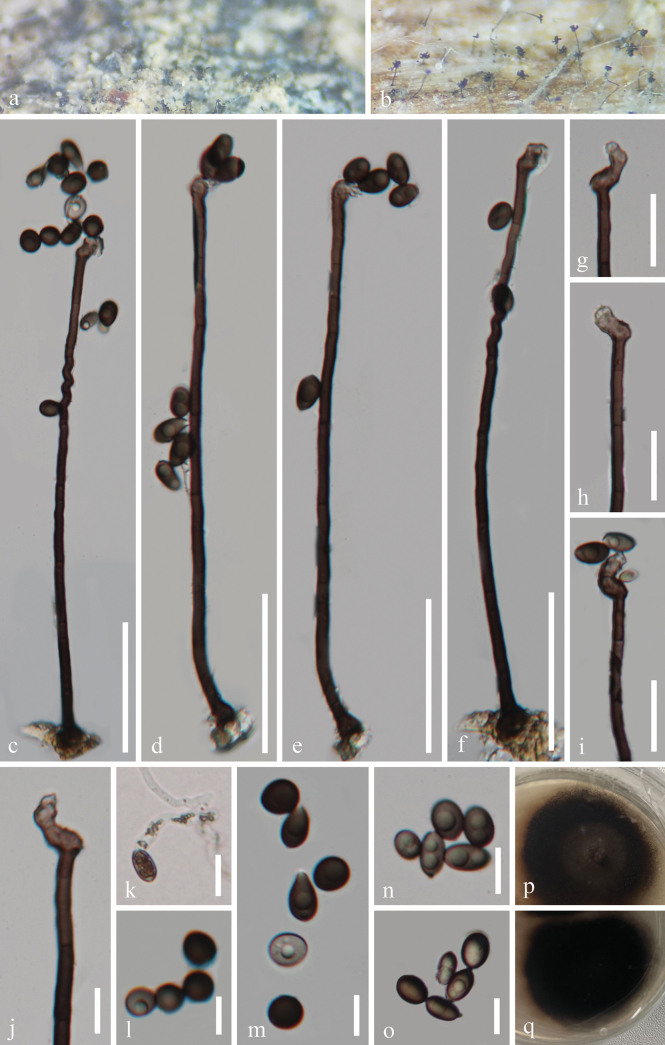
*Nigrellomyces aquaticus* (GZAAS 25-0660, holotype). a, b. Colonies on the host surface; c–f. Conidiophores, conidiogenous cells, and conidia; g–h. Conidiogenous cells; i-j. Conidiogenous cells and conidia; k. Germinated conidium; l–o. Conidia; p, q. Colonies on PDA from above and below after 41 days of incubation at room temperature. Scale bars: 60 μm (d–f); 50 μm (c); 20 μm (g–j); 10 μm (k–o).

#### Holotype.

GZAAS 25-0660

#### Description.

***Saprobic*** on decaying wood submerged in a freshwater stream. **Asexual morph: *Colonies*** superficial, effuse, hairy, brown to black, scattered, with glistening conidial masses at apex. ***Mycelium*** partly superficial, partly immersed, consisting of branched, septate, smooth, smooth-walled, hyaline to pale brown hyphae. ***Conidiophores*** macronematous, mononematous, solitary, erect, unbranched, multi-septate, straight, or flexuous, cylindrical, slightly constricted at septa, 146–364 × 4.5–8 μm *x ¯* = 224 × 5.8 μm, n = 25), dark brown, becoming pale brown to brown towards the apex. ***Conidiogenous cells*** polyblastic, integrated, terminal or intercalary, sympodial proliferations, sometimes denticles, cylindrical, reniform, curved, and swollen at the apex, 8.5–28 × 4–6 μm *x ¯* = 20 × 5.2 μm, n = 25), pale brown to brown. ***Conidia*** acrogenous, subglobose to globose, ovoid to obovoid, or ellipsoidal, aseptate, occasionally forming chains in water, guttulate, 7.5–12.5 × 5–8.5 μm *x ¯* = 8.9 × 7 μm, n = 25), light brown or black, smooth-walled, sometimes have small apiculus at the base. **Sexual morph**: Undetermined.

#### Culture characteristics.

Conidia germinate on PDA within 17 hours, producing germ tubes from the conidial body. Colonies on PDA are irregular with a flat surface and undulate margin, reaching 38 cm in diameter after 41 days at room temperature (approximately 25 °C), and are brown to dark brown on both the surface and reverse sides.

#### Material examined.

China • Guizhou Province, Guiyang City, Baiyun District, Changpo Ling National Forest Park, on rotting wood in a freshwater habitat, 15 March 2025, Wang-Ming Zhang, QX13 (GZAAS 25-0660, holotype), ex-type living cultures GZCC 25-0630; • Ibid., QX13.1 (GZAAS 25-0661, paratype), living culture GZCC 25-0631.

#### Notes.

In our phylogenetic tree (Fig. 1), isolates (GZCC 25-0630 and GZCC 25-0631) formed a sister clade to *Neomonodictys aquatica* (KUNCC 21-10708) and *N. muriformis* (MFLUCC 16-1136) with weak support. *Nigrellomyces aquaticus* (GZAAS 25-0660) can be distinguished from *Neomonodictys aquatica* (KUN-HKAS 115806) and *N. muriformis* (MFLU 17-1178) by its longer conidiophores, elongated, polyblastic conidiogenous cells, and aseptate conidia (Hyde et al. 2020; Huang et al. 2022). Moreover, base pair comparison of *Nigrellomyces aquaticus* (GZCC 25-0630) and *N. muriformis* (MFLUCC 16-1136) shows 96/588 bp differences in ITS (16.3%, gaps 37 bp) and 22/829 bp differences in LSU (2.7%, gap one bp). Therefore, based on multigene phylogenetic analysis and morphological differences, we introduce *Nigrellomyces aquaticus* as a novel genus and species.

## ﻿Discussion

The newly introduced genus *Nigrellomyces* shares morphological similarities with *Cordana*, including brown, septate conidiophores; polyblastic, swollen conidiogenous cells; and brown, obovoid to ellipsoidal conidia (Hughes 1955; Hernández-Restrepo et al. 2014; Hernandez Restrepo et al. 2015; Kuo et al. 2024; Xu et al. 2024b). However, *Nigrellomyces* differs from *Cordana* in several key features. Notably, its conidiogenous cells are curved at the apex and occasionally bear denticles, while its conidia are aseptate, in contrast to the typically septate conidia of *Cordana* (Hughes 1955; Hernández-Restrepo et al. 2014; Hernandez Restrepo et al. 2015; Kuo et al. 2024; Xu et al. 2024b). In addition to these morphological distinctions, the two genera are phylogenetically unrelated. *Cordana* is classified within the family Cordanaceae (Coniochaetales, Sordariomycetidae), whereas *Nigrellomyces* is placed in Pleurotheciaceae (Pleurotheciales, Savoryellomycetidae). Our multilocus phylogenetic analysis revealed that *Nigrellomyces* forms a monophyletic clade with strong support, positioned as a sister group to *Neomonodictys* within Pleurotheciaceae. Morphologically, *Nigrellomyces* is distinct from other genera in the family. While most members of Pleurotheciaceae exhibit acrodictys-like (Yanna and Hyde 2002; Sadowski et al. 2012), helicoön-like (Dayarathne et al. 2019; Réblová et al. 2020), monodictys-like (Hyde et al. 2020), and dactylaria-like (Réblová et al. 2016; Luo et al. 2018a) asexual morphs, *Nigrellomyces* displays unique cordana-like characters. Given its morphological and phylogenetic uniqueness, *Nigrellomyces* is established here as a new genus to accommodate this newly discovered taxon.

## Supplementary Material

XML Treatment for Nigrellomyces

XML Treatment for Nigrellomyces aquaticus

## ﻿Additional information

### Use of AI

No use of AI was reported.

### Data availability

All of the data that support the findings of this study are available in the main text.
